# The effects of brain radiotherapy combined with immunotherapy and chemotherapy for driver gene-negative non-small-cell lung cancer with brain metastases

**DOI:** 10.3389/fonc.2026.1763685

**Published:** 2026-07-06

**Authors:** Xiaoyu Zhang, Jiayi Huang, Xuan Liang, Zhiping Ruan, Yu Yao, Xiao Fu, Tao Tian

**Affiliations:** Department of Medical Oncology, The First Affiliated Hospital of Xi’an Jiaotong University, Xi’an, Shaanxi, China

**Keywords:** driver gene-negative non-small- cell lung cancer, brain metastases, immunotherapy, brain radiotherapy, chemotherapy

## Abstract

**Objective:**

Brain metastases (BM) are highly prevalent in advanced non-small cell lung cancer (NSCLC); however, therapeutic options remain limited and suboptimal for driver gene-negative NSCLC BM patients. This study aimed to explore the efficacy and prognostic risk factors of brain radiotherapy combined with immunotherapy and chemotherapy in real-world for these patients.

**Methods:**

This study included 222 driver gene-negative NSCLC patients with BM. Inverse Probability of Treatment Weighting (IPTW) was employed to reduce the impact of selection bias and confounding factors. Prognostic risk factors were identified using Cox regression analysis. Survival outcomes were evaluated using Kaplan–Meier curves, and subgroup analyses were performed to explore the associations between treatment and survival results.

**Results:**

Based on the use of immunotherapy, patients were divided into the RT+CT+ICI group (received brain radiotherapy combined with immunotherapy and chemotherapy, n=117) and the RT+CT group (received brain radiotherapy combined with chemotherapy alone, n=105). The median overall survival (mOS) longer in the RT+CT+ICI group (19.6 vs. 15 months, Hazard Ratio [HR] 0.57, 95% confidence interval [CI] 0.41–0.79, *P* < 0.001). Multivariable adjustments confirmed prolonged OS (HR 0.58, 95% CI 0.41–0.81, *P* = 0.002) and progression-free survival (PFS) (HR 0.72, 95% CI 0.53–0.98, *P* = 0.038) in the RT+CT+ICI group. Stereotactic radiosurgery (SRS) is a protective factor for OS in patients with driver gene-negative NSCLC BM; while PD-L1 expression is a protective factor for PFS and intracranial progression-free survival (IPFS) in these patients.

**Conclusions:**

This study demonstrates that brain radiotherapy combined with immunotherapy and chemotherapy is effective and predictive in driver gene-negative NSCLC BM patients. In addition, SRS and PD-L1 expression may be associated with better prognosis in such patients.

## 1 Introduction

The brain is one of the most prevalent sites for distant metastasis in advanced-stage lung cancer (1). Approximately 20% of patients diagnosed with NSCLC present with BM at the initial diagnosis, while 25%–50% will develop BM during disease progression (2). Once BM occur, patients typically have a poor overall prognosis (3). Despite the identification of multiple driver gene variants in lung cancer, a subset of NSCLC patients either lacks detectable driver genes or harbors rare mutation sites without available targeted therapeutic strategies. This is defined as driver gene-negative NSCLC (4). Targeted therapy has significantly enhanced the prognostic outcomes of NSCLC patients with BM who harbor actionable driver mutations (5). In contrast, patients with driver gene-negative NSCLC fail to benefit from targeted treatments. The effectiveness of conventional therapeutic approaches, including surgical resection, whole-brain radiotherapy (WBRT), and stereotactic radiotherapy (SRS), remains restricted (6). Chemotherapy is difficult to control effectively, primarily due to poor blood-brain barrier penetration, drug efflux pumping effects, and genetic heterogeneity between primary and metastases (7, 8).

The emergence of immunotherapy has reshaped the therapeutic landscape of advanced NSCLC (9, 10). Currently, immunotherapy combined with chemotherapy has become the standard first-line treatment strategy for patients with driver gene-negative NSCLC (11), offering new prospects for managing BM. However, due to the intricate nature of brain metastases, the brain’s distinctive microenvironment (12), challenges in tissue sample acquisition, and the strict inclusion criteria in earlier classic clinical studies on immunotherapy for advanced NSCLC, patients with active brain metastases are often excluded from clinical trials (8). Therefore, the effectiveness of immunotherapy in this specific patient population remains uncertain.

Prior research has demonstrated that the combination of brain radiotherapy and immunotherapy can extend survival duration, enhance the local control rate of intracranial lesions, and reduce the incidence of new intracranial lesions (13, 14). The positive immune activation effect, negative immune suppression effect, and distant effect of radiotherapy provide a theoretical basis for the combined application of these two modalities (15, 16). Therefore, the combination of radiotherapy and immunotherapy has emerged as a promising therapeutic strategy for patients with driver gene-negative NSCLC BM; however, there is no uniform consensus on the specific clinical application of this combination.

Previous phase III clinical studies often excluded patients with active symptomatic BM, resulting in limited treatment data for this patient. Therefore, this study aims to provide real-world data for the treatment of such patients and better assist clinical decision-making by evaluating the efficacy of brain radiotherapy combined with immunotherapy in patients with driver gene-negative NSCLC BM.

## 2 Methods

### 2.1 Patients enrolled

This retrospective study included 222 driver gene-negative NSCLC patients with BM from The First Affiliated Hospital of Xi’an Jiaotong University between December 2015 and December 2023.

**Figure 1:**
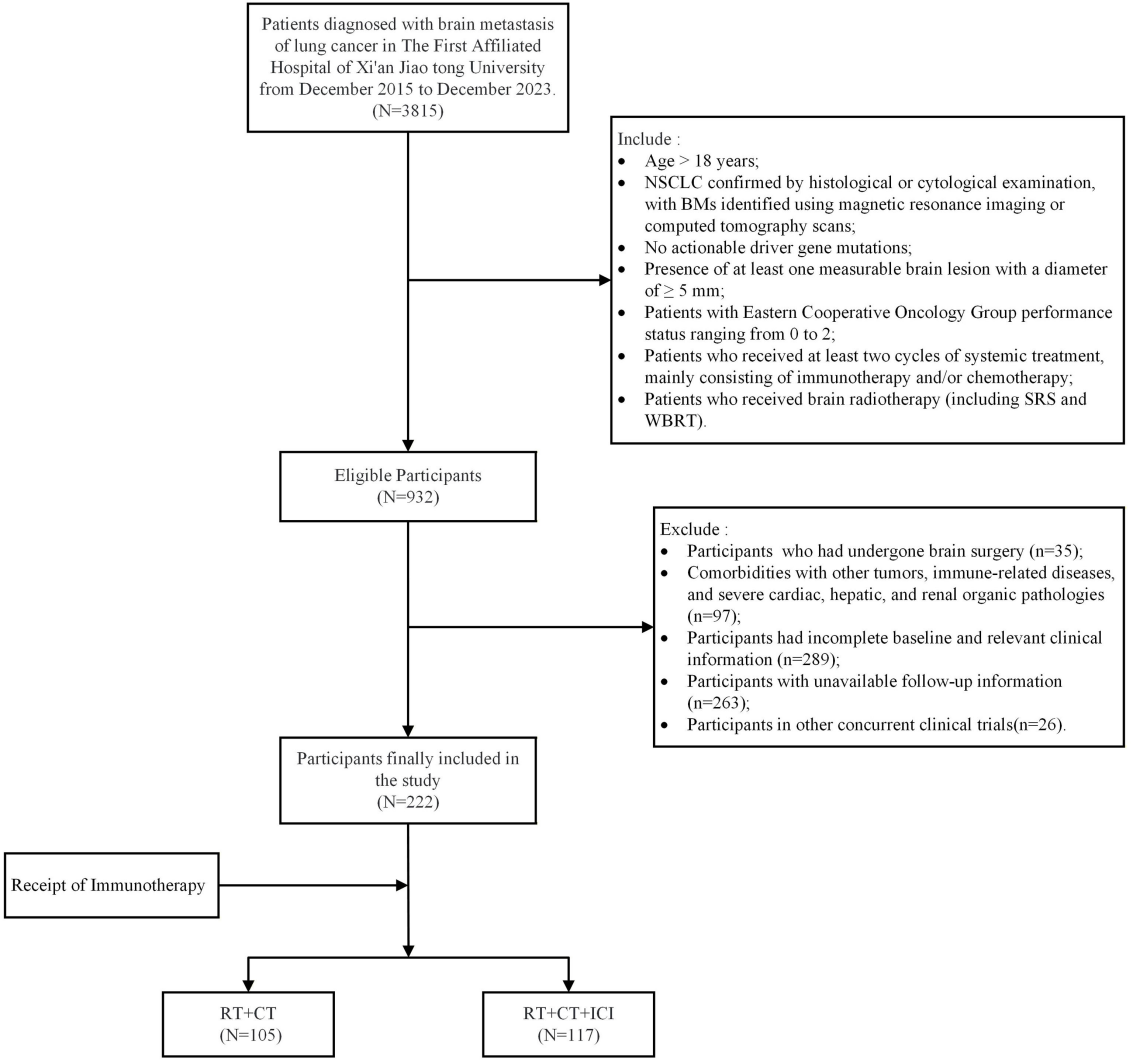
Flow chart of eligible participants selection. RT, radiotherapy; CT, chemotherapy; ICI, Immune checkpoint inhibitor; NSCLC, Non-small-cell lung cancer.

The patients included in this study met the following criteria: (1) Age > 18 years; (2) NSCLC confirmed by histological or cytological examination, with BM identified using magnetic resonance imaging or computed tomography scans; (3) No actionable driver gene mutations (e.g., EGFR, ALK, and ROS1, etc.); (4) Presence of at least one measurable brain lesion with a diameter of ≥ 5 mm; (5) Patients with Eastern Cooperative Oncology Group (ECOG) performance status ranging from 0 to 2; (6) Patients who received at least two cycles of systemic treatment, mainly consisting of immunotherapy and/or chemotherapy; (7) Patients who received brain radiotherapy (including SRS and WBRT). The exclusion criteria were as follows: (1) patients who had undergone brain surgery. (2) Comorbidities with other tumors, immune-related diseases, and severe cardiac, hepatic, and renal organic pathologies; (3) Participants had incomplete baseline and relevant clinical information; (4) Participants with unavailable follow-up information; (5) Participants in other concurrent clinical trials. Prior to 2016, routine testing for PD-L1 status was not performed on NSCLC samples (17). Therefore, we did not set strict PD-L1 level criteria for this study. The selection process is illustrated in the flowchart (**Figure 1**).

### 2.2 Demographic and clinicopathological data collection

Demographic data were extracted, including age, sex, and history of smoking, hypertension, diabetes, ECOG, and Graded Prognostic Assessment (GPA). The clinicopathological data included the following: tumor information, histopathology, TNM stage, the number of BM, location of BM, clinical presentation (symptomatic vs. asymptomatic), the number of extracranial organ metastases (ECM), temporal heterogeneity of BM, and PD-L1 expression status.

In this study, the variables were categorized according to their clinical significance. Based on the smoking index (SI), patients were classified as never smokers (0), light smokers (≤200), moderate smokers (200-400), and heavy smokers (≥400). A GPA score of 3.5–4 is considered a good prognosis; 2.5–3 indicates a moderate prognosis; 1.5–2 indicates a poor prognosis; and 0–1 indicates a dismal prognosis. Synchronous brain metastases (SBM) were defined as BMs diagnosed within 2 months of primary cancer diagnosis, and metachronous brain metastases (MBM) were defined as primary cancer and metastasis diagnoses separated by at least 2 months (18).

### 2.3 Treatments

Therapeutic data included brain radiotherapy, ICIs, and chemotherapy regimens. The selection of brain radiotherapy modalities and doses is based on the number of intracranial lesions in individual patients. SRS was administered at 24–35 Gy in 3–5 fractions or as a single dose of 18–22 Gy, based on the patient’s lesion volume. WBRT was delivered in 2–3 Gy per fraction, once daily, for 10–20 fractions, with a total dose of 30–40 Gy. Chemotherapy regimens were determined according to the patients’ pathological types and conditions, mainly including platinum-based dual-drug chemotherapy and monotherapies. For the combination of cranial radiotherapy with immunotherapy and chemotherapy (within 1 month before or after radiotherapy), the selection of radiotherapy and chemotherapy regimens remains the same as those mentioned above. Immunotherapy included the use of anti-PD-1 or anti-PD-L1 antibodies, such as Atezolizumab, Camrelizumab, Envafolimab, Pembrolizumab, Sintilimab, Tislelizumab, and Toripalimab, with a total course of 2–8 cycles.

### 2.4 Patients’ follow-up and study endpoint

All patients underwent regular follow-up through electronic medical records and telephone communication. The cohort had a median follow-up of 43.5 months (95% CI, 37.1–45.8) until April 30, 2025. Of these, 155 patients died, and 67 were censored due to loss to follow-up (censored).

Efficacy assessments in this study were based on the Response Evaluation Criteria in Solid Tumors (RECIST), version 1.1 (19). The primary endpoint was overall survival (OS), defined as the time from BM diagnosis to death from any cause. The secondary endpoints included progression-free survival (PFS; time from BM diagnosis to systemic progression or death) and intracranial PFS (IPFS; time from BM diagnosis to intracranial progression).

### 2.5 Statistics analysis

Continuous variables were transformed into categorical variables based on their clinical relevance, and categorical variables were presented as counts and percentages. Group differences were compared using Pearson’s chi-square test or Fisher’s exact test, as appropriate. IPTW was used to reduce bias in baseline characteristics, with a standardized mean difference (SMD) greater than 0.1 indicating covariate imbalance. Survival curves were constructed using Kaplan-Meier analysis, and between-group differences were analyzed using the log-rank test. The Reverse Kaplan-Meier analysis was used to estimate the median follow-up time. Univariate and multivariate Cox regression analyses were performed to identify prognostic risk factors. After adjusting for potential confounding variables, subgroup analyses were performed to evaluate the association between treatment characteristics and survival outcomes. Interaction tests within Cox proportional hazards models were employed to compare hazard ratios (HR) across different subgroups.

All statistical tests in this study were two-tailed, with statistical significance determined using a threshold of *P* < 0.05. Statistical analyses were performed using R software (version 4.3.3).

## 3 Result

### 3.1 Baseline and survival characteristics

A total of 222 driver gene-negative NSCLC patients with BM were enrolled in this study. Based on the use of immunotherapy, patients were divided into two groups: the RT+CT+ICI group (n=117, received brain radiotherapy combined with immunotherapy and chemotherapy) and the RT+CT group (n=105, received brain radiotherapy combined with chemotherapy alone). When calculating the SMD, we observed minimal between-group differences (SMD < 0.1) (**Supplementary File 1**). This indicates well-balanced baseline characteristics and comparable groups. Other clinical information is presented in **Table 1**. Owing to missing values in PD-L1 expression level, IPTW was not performed. And the *P*-value < 0.01 indicated potential between-group differences in PD-L1 expression level, which will be further analyzed.

**Table 1:** Baseline characteristics of system therapy.

Variable		RT+CT (N = 105)	RT+CT+ICI (N = 117)	*P*-value^1^
Age	<65	78 (74%)	82 (70%)	0.486
	≥65	27 (26%)	35 (30%)	
Sex	Female	22 (21%)	16 (14%)	0.151
	Male	83 (79%)	101 (86%)	
SI	Heavy smoking	57 (54%)	53 (45%)	0.402
	Light smoking	6 (6%)	12 (10%)	
	Moderate smoking	4 (4%)	3 (3%)	
	never smoke	38 (36%)	49 (42%)	
Hypertension	No	77 (73%)	93 (79%)	0.280
	Yes	28 (27%)	24 (21%)	
Diabetes	No	94 (90%)	105 (90%)	0.957
	Yes	11 (10%)	12 (10%)	
ECOG	0	4 (4%)	3 (2%)	0.896
	1	92 (88%)	104 (89%)	
	2	9 (8%)	10 (9%)	
GPA	Dismal	26 (25%)	25 (21%)	0.200
	Good	6 (6%)	15 (13%)	
	Moderate	39 (37%)	34 (29%)	
	Poor	34 (32%)	43 (37%)	
Histopathology	LUAD	78 (74%)	87 (74%)	0.990
	LUSC	27 (26%)	30 (26%)	
BM lesion	All	32 (30%)	38 (33%)	0.945
	Infratentorial BM	19 (18%)	20 (17%)	
	Supratentorial BM	54 (52%)	59 (50%)	
T stage	1	11 (10%)	13 (11%)	0.238
	2	45 (43%)	45 (39%)	
	3	30 (29%)	25 (21%)	
	4	19 (18%)	34 (29%)	
N stage	0	23 (22%)	16 (14%)	0.336
	1	23 (22%)	26 (22%)	
	2	41 (39%)	47 (40%)	
	3	18 (17%)	28 (24%)	
PD-L1 (TPS)	<1%	29 (28%)	24 (20%)	<0.001
	≥1%	16 (15%)	51 (44%)	
	Unknown	60 (57%)	42 (36%)	
No. of BM	>3	45 (43%)	47 (40%)	0.685
	≤3	60 (57%)	70 (60%)	
No. of ECM	≥2	22 (21%)	25 (21%)	0.912
	0	47 (45%)	55 (47%)	
	1	36 (34%)	37 (32%)	
Clinical presentation	Asymptomatic	44 (42%)	63 (54%)	0.075
	Symptomatic	61 (58%)	54 (46%)	
Temporal specificity	MBM	53 (50%)	54 (46%)	0.520
	SBM	52 (50%)	63 (54%)	
RT method	SRS	82 (78%)	102 (87%)	0.073
	WBRT	23 (22%)	15 (13%)	

^1^ Pearson’s Chi-squared test or Fisher’s exact test.

ICI, Immune checkpoint inhibitors; CT, chemotherapy; RT, brain radiotherapy; BM, Brain metastases; SI, Smoking Index; ECOG, Eastern Cooperative Oncology Group; GPA, Graded Prognostic Assessment; LUAD, Lung adenocarcinoma; LUSC, Lung squamous cell carcinoma; ECM, Extracranial metastasis; PD-L1, Programmed cell death 1 ligand 1; TPS, Tumor Proportion Score; SBM, Synchronous brain metastasis; MBM, Metachronous brain metastasis; WBRT, whole-brain radiation therapy; SRS, stereotactic radiosurgery.

SI: never smoke (0); Light smoking (≤200); Moderate smoking (200-400); Heavy smoking (≥400). GPA: 3.5–4 Good prognosis; 2.5–3 Moderate prognosis; 1.5–2 Poor prognosis; 0–1 Dismal prognosis. Synchronous is defined as BM diagnosis within 2 months of primary cancer diagnosis; Metachronous diagnosis is defined as primary cancer and metastasis diagnosis separated by at least 2 months.

**Figure 2:**
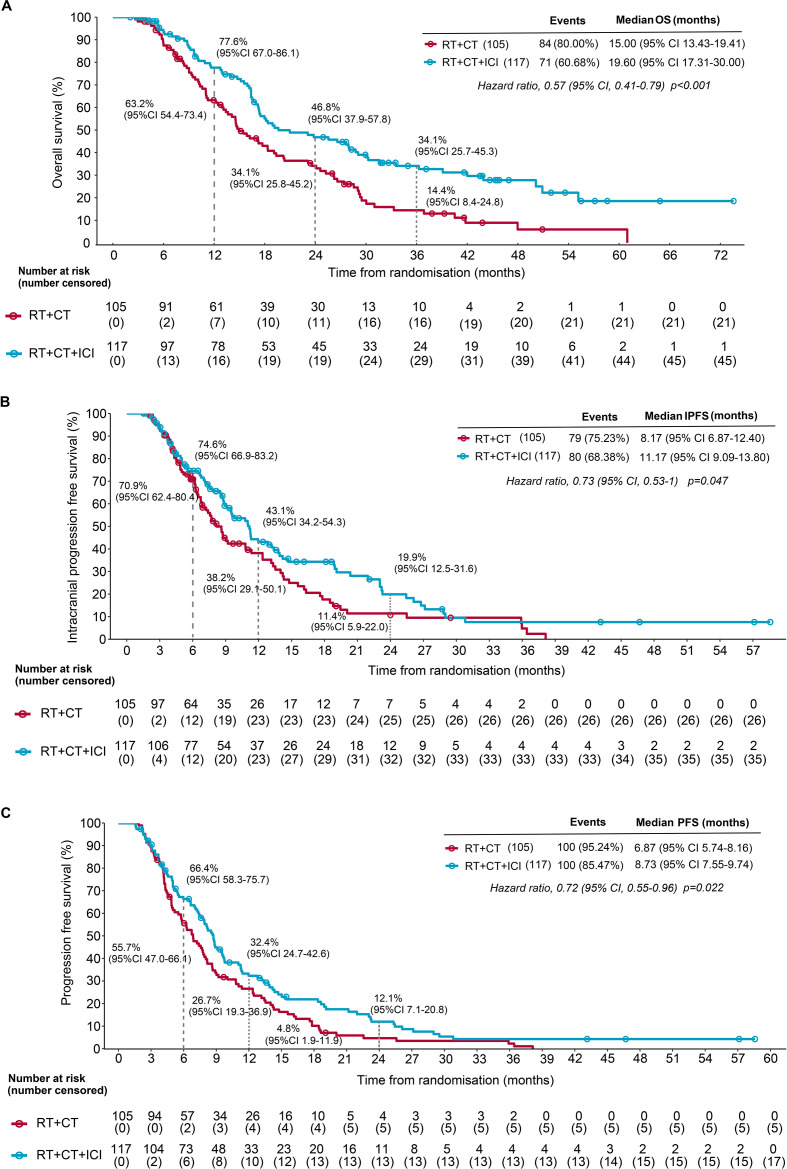
Kaplan–Meier curves for system therapy (RT+CT+ICI vs. RT+CT). **(A)** OS of the two groups; **(B)** IPFS of the two groups; **(C)** PFS of the two groups. RT, radiotherapy; CT, chemotherapy; ICI, Immune checkpoint inhibitor; IPFS, Intracranial progression-free survival; OS, Overall survival; PFS, Progression-free survival.

The mOS was 15.0 months (95% CI 13.43–19.41) for patients in the CT+RT group and 19.6 months (95% CI 17.31–30.00) for patients in the RT+CT+ICI group, showing a significant difference (HR 0.57, 95%CI 0.41–0.79, *P* < 0.001, **Figure 2A**). The mIPFS was 8.17 months (95% CI 6.87–12.40) for patients in the CT+RT group and 11.17 months (95% CI 9.09–13.80) for patients in the RT+CT+ICI group (HR 0.73, 95%CI 0.53–1, *P* = 0.047, **Figure 2B**). The mPFS in the CT+RT group was 6.87 months (95% CI 5.74–8.16), whereas in the RT+CT+ICI group, it was 8.73 months (95% CI 7.55–9.74) (HR 0.72, 95%CI 0.55–0.96, *P* = 0.022, **Figure 2C**). The results indicated that compared with non-combined immunotherapy, brain radiotherapy combined with immunotherapy and chemotherapy could significantly prolong the OS, PFS, and IPFS of driver-negative NSCLC BM patients.

### 3.2 Associations of brain radiotherapy combined with immunotherapy and survival

Next, we gradually adjusted the confounding factors to further explore the relationship between combined immunotherapy and survival. In the initial analysis, patients in the RT+CT+ICI group showed a better prognosis than those in the RT+CT group (OS: HR 0.57, 95% CI 0.41–0.79, *P* < 0.001; PFS: HR 0.72, 95% CI 0.55–0.96, *P* = 0.022; IPFS: HR 0.73, 95% CI 0.53–1.00, *P* = 0.048). In multivariable adjusted model 1, controlling for age, sex, SI, hypertension, diabetes, and ECOG, the OS (HR 0.53, 95% CI 0.38–0.74, *P* < 0.001) and PFS (HR 0.70, 95% CI 0.52–0.94, *P* = 0.016) remained consistent in the RT+CT+ICI group. In multivariable model 2, which included additional adjustments for histopathology, BM lesion, T stage, N stage, the number of BMs, the number of ECM, symptoms of BM, and temporal heterogeneity, the findings remained significant in OS (HR 0.58, 95% CI 0.41–0.81, *P* = 0.002) and PFS (HR 0.72, 95% CI 0.53–0.98, *P* = 0.038). However, the two groups showed no difference in IPFS (*P* = 0.199). Overall, these findings indicate that RT+CT+ICI is associated with a notably prolonged OS and PFS compared with RT+CT (**Table 2**).

**Table 2:** Associations between systemic therapy and clinical outcomes.

Systemic therapy	Univariable model		Multivariable model 1		Multivariable model 2	
	HR (95% CI)	*P* value	HR (95% CI)	*P* value	HR (95% CI)	*P* value
OS						
RT+CT	1.00 (Reference)		1.00 (Reference)		1.00 (Reference)	
RT+CT+ICI	0.57 (0.41, 0.79)	<0.001	0.53 (0.38, 0.74)	<0.001	0.58 (0.41, 0.81)	0.002
PFS						
RT+CT	1.00 (Reference)		1.00 (Reference)		1.00 (Reference)	
RT+CT+ICI	0.72 (0.55, 0.95)	0.022	0.70 (0.52, 0.94)	0.016	0.72 (0.53, 0.98)	0.038
IPFS						
RT+CT	1.00 (Reference)		1.00 (Reference)		1.00 (Reference)	
RT+CT+ICI	0.73 (0.53, 1.00)	0.048	0.77 (0.56, 1.07)	0.115	0.80 (0.57, 1.13)	0.199

Multivariable model 1 was adjusted for age, sex, SI, hypertension, diabetes, and ECOG.

Multivariable model 2 was additional adjustments for histopathology, BM lesion, T stage, N stage, no. of BM, no. of ECM, clinical presentation, and temporal specificity.

CI, Confidence Interval; HR, Hazard Ratio; OS, overall survival; PFS, progression free survival; IPFS, intracranial progression free survival.

### 3.3 Subgroup analysis and interaction effects of two groups

Subgroup analyses were performed in different subgroups to evaluate possible effect modifications in the association between therapy and prognosis. According to the subgroup analysis, the RT+CT+ICI group had better OS, PFS, and IPFS in driver gene-negative NSCLC with BM compared with the RT+CT group (**Supplementary File 2**). There was a significant interaction effect between the number of ECM and treatment (*P* for interaction = 0.018) in the OS analyses (**Supplementary File 2A**). In the number of ECMs≥2, the effect did not reach statistical significance (HR 1.32, 95% CI 0.63–2.77, *P* = 0.464). In contrast, when the number of ECM was 0 and 1, the protective effect of RT+CT+ICI was significant (No. of ECMs=0: HR 0.42, 95% CI 0.27–0.68, *P* < 0.001; No. of ECMs=1: HR 0.50, 95% CI 0.28–0.88, *P* =0.017). In the subgroup analyses of PFS and IPFS, a strong interaction effect was observed between treatment and GPA score (IPFS: *P* for interaction = 0.007; PFS: *P* for interaction = 0.038; **Supplementary Files 2B, C**). Additionally, the interaction effect of treatment on OS, PFS, and IPFS did not significantly differ across subgroups based on other variables.

### 3.4 Association of radiotherapy modalities and clinical outcomes

To further explore the relationship between different radiotherapy modalities and survival, we performed IPTW on the SRS and WBRT groups to eliminate inter-group differences; multiple indicators differed pre-IPTW, while all post-IPTW SMDs were < 0.1, indicating balanced and comparable baseline data (**Supplementary File 3**). Other clinical characteristic are presented in **Table 3**.

**Table 3:** Clinical characteristics of radiotherapy modalities.

Variable		SRS (N = 184)	WBRT (N = 38)	*P*-value^1^
Age	<65	133 (72%)	27 (71%)	0.878
	≥65	51 (28%)	11 (29%)	
Sex	Female	32 (17%)	6 (16%)	0.811
	Male	152 (83%)	32 (84%)	
SI	Heavy smoking	91 (49%)	19 (50%)	0.618
	Light smoking	14 (7.6%)	4 (11%)	
	Moderate smoking	5 (2.7%)	2 (5.3%)	
	never smoke	74 (40%)	13 (34%)	
Hypertension	No	139 (76%)	31 (82%)	0.424
	Yes	45 (24%)	7 (18%)	
Diabetes	No	163 (89%)	36 (95%)	0.383
	Yes	21 (11%)	2 (5.3%)	
ECOG	0	7 (3.8%)	0 (0%)	0.503
	1	160 (87%)	36 (95%)	
	2	17 (9.2%)	2 (5.3%)	
GPA	Dismal	40 (22%)	11 (29%)	0.708
	Good	19 (10%)	2 (5.3%)	
	Moderate	61 (33%)	12 (32%)	
	Poor	64 (35%)	13 (34%)	
Histopathology	LUAD	136 (74%)	29 (76%)	0.758
	LUSC	48 (26%)	9 (24%)	
BM lesion	All	52 (28%)	18 (47%)	0.026
	Infratentorial BM	31 (17%)	8 (21%)	
	Supratentorial BM	101 (55%)	12 (32%)	
T stage	1	17 (9.2%)	7 (18%)	0.090
	2	73 (40%)	17 (45%)	
	3	45 (24%)	10 (26%)	
	4	49 (27%)	4 (11%)	
N stage	0	29 (16%)	10 (26%)	0.333
	1	40 (22%)	9 (24%)	
	2	74 (40%)	14 (37%)	
	3	41 (22%)	5 (13%)	
PD-L1 (TPS)	<1%	46 (45%)	7 (41%)	0.789
	≥1%	57 (55%)	10 (59%)	
	Unknown	81	21	
No. of BM	>3	67 (36%)	25 (66%)	<0.001
	≤3	117 (64%)	13 (34%)	
No. of ECM	≥2	40 (22%)	7 (18%)	0.820
	0	85 (46%)	17 (45%)	
	1	59 (32%)	14 (37%)	
Clinical presentation	Asymptomatic	93 (51%)	14 (37%)	0.124
	Symptomatic	91 (49%)	24 (63%)	
Temporal specificity	MBM	89 (48%)	18 (47%)	0.910
	SBM	95 (52%)	20 (53%)	
System therapy	RT+CT	82 (45%)	23 (61%)	0.073
	RT+CT+ICI	102 (55%)	15 (39%)	

^1^ Pearson’s Chi-squared test or Fisher’s exact test.

ICI, Immune checkpoint inhibitors; CT, chemotherapy; RT, brain radiotherapy; BM, Brain metastases; SI, Smoking Index; ECOG, Eastern Cooperative Oncology Group; GPA, Graded Prognostic Assessment; LUAD, Lung adenocarcinoma; LUSC, Lung squamous cell carcinoma; ECM, Extracranial metastasis; PD-L1, Programmed cell death 1 ligand 1; TPS, Tumor Proportion Score; SBM, Synchronous brain metastasis; MBM, Metachronous brain metastasis; WBRT, whole-brain radiation therapy; SRS, stereotactic radiosurgery.

SI: never smoke (0); Light smoking (≤200); Moderate smoking (200-400); Heavy smoking (≥400). GPA: 3.5–4 Good prognosis; 2.5–3 Moderate prognosis; 1.5–2 Poor prognosis; 0–1 Dismal prognosis. Synchronous is defined as BM diagnosis within 2 months of primary cancer diagnosis; Metachronous diagnosis is defined as primary cancer and metastasis diagnosis separated by at least 2 months.

**Figure 3:**
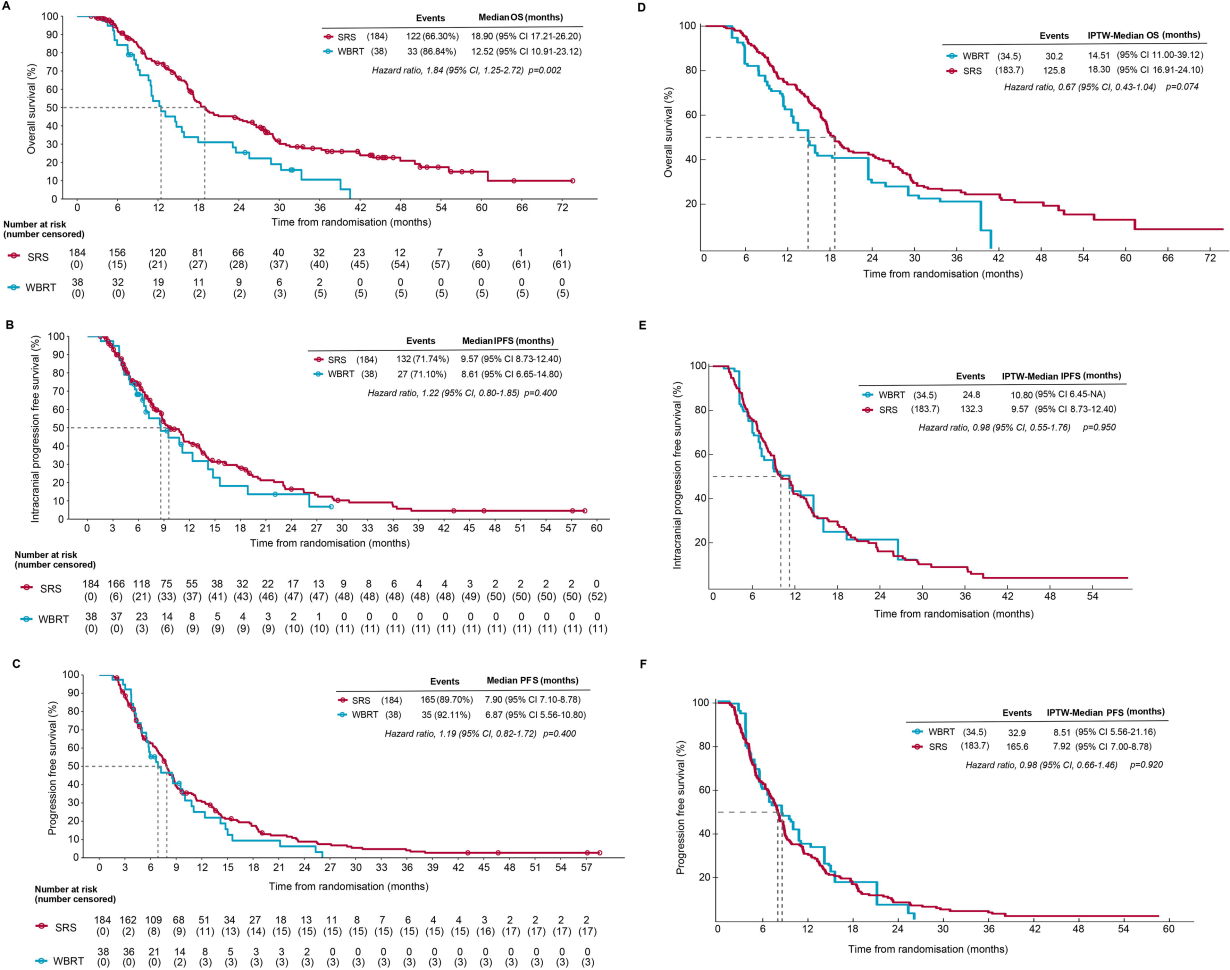
Kaplan–Meier curves of radiotherapy modalities before and after IPTW. **(A)** OS of patients with different radiotherapy modalities before IPTW; **(B)** IPFS of patients with different radiotherapy modalities before IPTW; **(C)** PFS of patients with different radiotherapy modalities before IPTW; **(D)** OS of patients with different radiotherapy modalities after IPTW; **(E)** IPFS of patients with different radiotherapy modalities after IPTW; **(F)** PFS of patients with different radiotherapy modalities after IPTW. SRS, stereotactic radiosurgery; WBRT, whole-brain radiotherapy; IPFS, Intracranial progression-free survival; OS, Overall survival; PFS, Progression-free survival.

Before IPTW, the OS of WBRT group was shorter than SRS group, and this difference was statistically significant (HR1.84, 95% CI 1.25–2.72, *P* = 0.002) (**Figure 3A**). However, this difference disappeared after IPTW (**Figure 3D**) and there was no difference in IPFS (**Figures 3B, E**) or IPFS (**Figures 3C, F**) between the two groups. This may be related to the large disparity in patient numbers between the two groups. After adjusting for all confounding factors, we found that SRS demonstrated a significant protective effect on OS (multivariable model 2: HR 0.59, 95% CI 0.35–0.99, *P* = 0.047). However, whatever unadjusted or adjusted for confounders, the effect was not significant for PFS and IPFS (**Table 4**).

**Table 4:** Associations between radiotherapy modalities and clinical outcomes.

Radiotherapy modalities	Univariable model		Multivariable model 1		Multivariable model 2	
	HR (95% CI)	*P* value	HR (95% CI)	*P* value	HR (95% CI)	*P* value
OS						
WBRT	1.00 (Reference)		1.00 (Reference)		1.00 (Reference)	
SRS	0.67 (0.43, 1.04)	0.075	0.67 (0.41, 1.09)	0.108	0.59 (0.35, 0.99)	0.047
PFS						
WBRT	1.00 (Reference)		1.00 (Reference)		1.00 (Reference)	
SRS	0.98 (0.66, 1.46)	0.919	0.94 (0.66, 1.33)	0.715	0.85 (0.58, 1.24)	0.476
IPFS						
WBRT	1.00 (Reference)		1.00 (Reference)		1.00 (Reference)	
SRS	0.98 (0.55, 1.76)	0.953	0.89 (0.56, 1.41)	0.609	0.94 (0.60, 1.49)	0.916

Multivariable model 1 was adjusted for age, sex, SI, hypertension, diabetes, and ECOG.

Multivariable model 2 was additional adjustments for histopathology, BM lesion, T stage, N stage, no. of BM, no. of ECM, clinical presentation, and temporal specificity.

SRS, stereotactic radiosurgery; WBRT, whole-brain radiotherapy; CI, Confidence Interval; HR, Hazard Ratio; OS, overall survival; PFS, progression free survival; IPFS, intracranial progression free survival.

### 3.5 Associations between PD-L1 expression and survival outcomes

Next, we explored the relationship between PD-L1 expression and survival in patients with driver gene-negative NSCLC BM, with all indicators being balanced and comparable after IPTW (**Supplementary File 4**). The clinical features are shown in **Table 5**.

**Table 5:** Clinical characteristics of PD-L1 expression.

Variable		PD-L1<1% (N = 53)	PD-L1≥1% (N = 67)	*P*-value^1^
Age	<65	35 (66%)	46 (69%)	0.761
	≥65	18 (34%)	21 (31%)	
Sex	Female	9 (17%)	9 (13%)	0.589
	Male	44 (83%)	58 (87%)	
SI	Heavy smoking	27 (51%)	35 (52%)	0.680
	Light smoking	6 (11%)	5 (7.5%)	
	Moderate smoking	1 (1.9%)	4 (6.0%)	
	never smoke	19 (36%)	23 (34%)	
Hypertension	No	41 (77%)	53 (79%)	0.818
	Yes	12 (23%)	14 (21%)	
Diabetes	No	46 (87%)	60 (90%)	0.640
	Yes	7 (13%)	7 (10%)	
ECOG	0	0 (0%)	2 (3.0%)	0.441
	1	50 (94%)	58 (87%)	
	2	3 (5.7%)	7 (10%)	
GPA	Dismal	15 (28%)	16 (24%)	0.182
	Good	3 (5.7%)	9 (13%)	
	Moderate	20 (38%)	16 (24%)	
	Poor	15 (28%)	26 (39%)	
Histopathology	LUAD	40 (75%)	53 (79%)	0.636
	LUSC	13 (25%)	14 (21%)	
BM lesion	All	18 (34%)	24 (36%)	0.450
	Infratentorial BM	7 (13%)	14 (21%)	
	Supratentorial BM	28 (53%)	29 (43%)	
T stage	1	2 (3.8%)	10 (15%)	0.240
	2	23 (43%)	25 (37%)	
	3	12 (23%)	15 (22%)	
	4	16 (30%)	17 (25%)	
N stage	0	5 (9.4%)	11 (16%)	0.355
	1	10 (19%)	13 (19%)	
	2	19 (36%)	28 (42%)	
	3	19 (36%)	15 (22%)	
radiotherapy	SRS	46 (87%)	57 (85%)	0.789
	WBRT	7 (13%)	10 (15%)	
No. of BM	>3	17 (32%)	29 (43%)	0.210
	≤3	36 (68%)	38 (57%)	
No. of ECM	≥2	12 (23%)	16 (24%)	0.985
	0	23 (43%)	29 (43%)	
	1	18 (34%)	22 (33%)	
Clinical presentation	Asymptomatic	26 (49%)	38 (57%)	0.404
	Symptomatic	27 (51%)	29 (43%)	
Temporal specificity	MBM	23 (43%)	33 (49%)	0.523
	SBM	30 (57%)	34 (51%)	
System therapy	RT+CT	29 (55%)	16 (24%)	<0.001
	RT+CT+ICI	24 (45%)	51 (76%)	

^1^ Pearson’s Chi-squared test or Fisher’s exact test.

ICI, Immune checkpoint inhibitors; CT, chemotherapy; RT, brain radiotherapy; BMs, Brain metastases; SI, Smoking Index; ECOG, Eastern Cooperative Oncology Group; GPA, Graded Prognostic Assessment; LUAD, Lung adenocarcinoma; LUSC, Lung squamous cell carcinoma; ECM, Extracranial metastasis; PD-L1, Programmed cell death 1 ligand 1; TPS, Tumor Proportion Score; SBM, Synchronous brain metastasis; MBM, Metachronous brain metastasis; WBRT, whole-brain radiation therapy; SRS, stereotactic radiosurgery.

SI: never smoke (0); Light smoking (≤200); Moderate smoking (200-400); Heavy smoking (≥400). GPA: 3.5–4 Good prognosis; 2.5–3 Moderate prognosis; 1.5–2 Poor prognosis; 0–1 Dismal prognosis. Synchronous is defined as BM diagnosis within 2 months of primary cancer diagnosis; Metachronous diagnosis is defined as primary cancer and metastasis diagnosis separated by at least 2 months.

**Figure 4:**
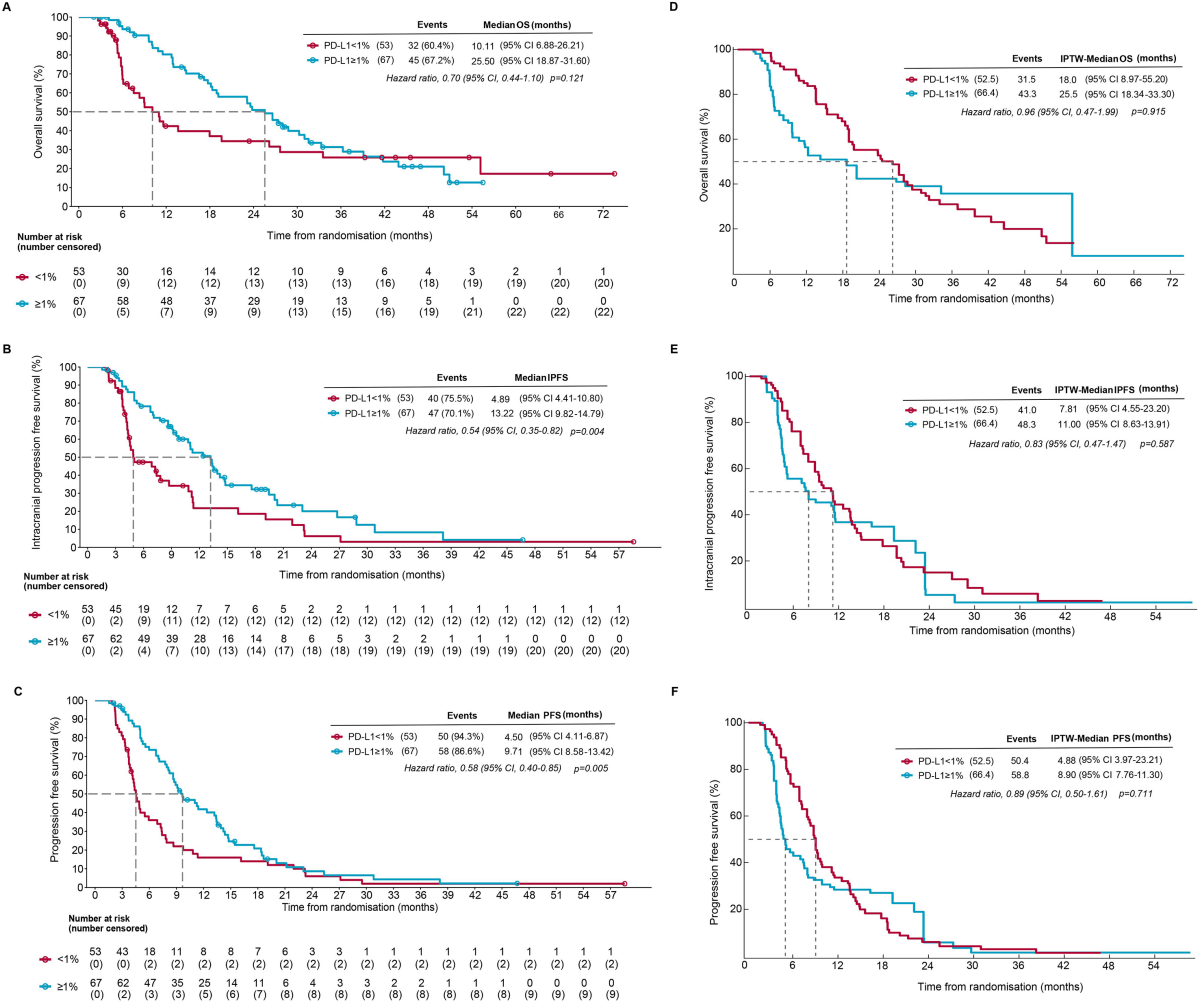
Kaplan–Meier curves of PD-L1 expression before and after IPTW. **(A)** OS of patients with different PD-L1 expression before IPTW; **(B)** IPFS of patients with different PD-L1 expression before IPTW; **(C)** PFS of patients with different PD-L1 expression before IPTW; **(D)** OS of patients with different PD-L1 expression after IPTW; **(E)** IPFS of patients with different PD-L1 expression after IPTW; **(F)** PFS of patients with different PD-L1 expression after IPTW. PD-L1, Programmed cell death 1 ligand 1; IPFS, Intracranial progression-free survival; OS, Overall survival; PFS, Progression-free survival.

Survival analysis showed that before IPTW, patients with PD-L1 expression had significantly longer IPFS and PFS than those without (**Figures 4B, C**); this difference disappeared after IPTW (**Figures 4E, F**). OS did not differ significantly across PD-L1 expression before and after IPTW (**Figures 4A, D**). However, after adjusting for all confounding factors, PD-L1 expression levels had a significant effect on PFS (multivariable model 2: HR 0.62, 95% CI 0.40–0.97, *P* = 0.038), and IPFS (multivariable model 2: HR 0.58, 95% CI 0.35–0.97, *P* = 0.037) (**Table 6**). This may be related to the fact that patients with PD-L1 expression can better benefit from immunotherapy.

**Table 6:** Associations between PD-L1 expression and clinical outcomes.

PD-L1 expression	Univariable model		Multivariable model 1		Multivariable model 2	
	HR (95% CI)	*P* value	HR (95% CI)	*P* value	HR (95% CI)	*P* value
OS						
<1%	1.00 (Reference)		1.00 (Reference)		1.00 (Reference)	
≥1%	0.96 (0.46, 1.99)	0.915	0.90 (0.45, 1.80)	0.768	0.67 (0.36, 1.26)	0.212
PFS						
<1%	1.00 (Reference)		1.00 (Reference)		1.00 (Reference)	
≥1%	0.89 (0.49, 1.61)	0.707	0.74 (0.44, 1.25)	0.258	0.62 (0.40, 0.97)	0.038
IPFS						
<1%	1.00 (Reference)		1.00 (Reference)		1.00 (Reference)	
≥1%	0.83 (0.47, 1.47)	0.522	0.89 (0.50, 1.57)	0.690	0.58 (0.35, 0.97)	0.037

Multivariable model 1 was adjusted for age, sex, SI, hypertension, diabetes, and ECOG.

Multivariable model 2 was additional adjustments for histopathology, BM lesion, T stage, N stage, no. of BM, no. of ECM, clinical presentation, and temporal specificity.

CI, Confidence Interval; HR, Hazard Ratio; OS, overall survival; PFS, progression free survival; IPFS, intracranial progression free survival.

## 4 Discussion

This study retrospectively analyzed the clinical characteristics and survival outcomes of 222 patients with driver-negative NSCLC BM. This study aimed to explore the efficacy of brain radiotherapy combined with immunotherapy and chemotherapy and identify the factors influencing prognosis in the real world.

Our findings demonstrated that the combination of brain radiotherapy, immunotherapy, and chemotherapy exhibits potential efficacy in improving prognosis among driver gene-negative NSCLC patients with BM. The study found that patients treated with brain radiotherapy combination with chemotherapy and immunotherapy achieved significant survival benefits compared with those who did not receive immunotherapy (mOS: 19.6m vs 15.0m; mIPFS: 11.17m vs 8.17m; mPFS: 8.73m vs 6.87m).The C-BRAIN trial confirmed the efficacy of first-line camrelizumab combined with chemotherapy and brain radiotherapy in NSCLC patients with brain metastases, with a mIPFS of 16.1 months and a mOS of 20.9 months (14).The CTONG2003 study also demonstrated that the camrelizumab combination with radiotherapy group had significantly longer mIPFS (19.1 vs 9.9) and mPFS (11.2 vs 6.7) than the placebo group (20). These findings indicate that immunotherapy combined with chemotherapy and radiotherapy yields promising efficacy in NSCLC patients with BM, which is consistent with the results of the present study. However, no restriction was imposed on the type of immunotherapeutic agents in this study. Therefore, the impact of different immune checkpoint inhibitors on patient survival warrants further investigation in future studies. Subgroup and interaction analyses demonstrated an interaction between OS and the number of metastases in extracranial organs. Patients with 0 or 1 ECM derived greater OS benefit from brain radiotherapy combined with immunotherapy than those with ≥2 ECMs, which is consistent with previous studies indicating that low extracranial tumor burden predicts superior OS with combined therapy (21–23). Moreover, significant interactions were identified between GPA score and both PFS and IPFS. Improved PFS and IPFS were more pronounced in patients with GPA 3.5–4 and 0–1. This may be attributed to better baseline performance status and enhanced radio-immunological synergy in patients with high GPA scores, while effective intracranial disease control underlies the benefit in those with low GPA scores (24, 25).Previous clinical studies have found radiotherapy to be more effective when combined with immunotherapy. This may be due to the immunomodulatory properties of radiotherapy and the synergistic effects of the two treatments (13). Remodeling the immunosuppressive TME inhibits tumor proliferation, enhances tumor sensitivity to radiotherapy, and boosts antitumor immunity (26). Radiotherapy induces immunogenic cell death, thereby facilitating T cell-mediated anti-tumor immune responses (27, 28). Through activation of the cGAS-STING signaling pathway, radiotherapy triggers type I interferon cascades that amplify anti-tumor immune responses (29–32). It upregulates the expression of MHC class I molecules on the surface of tumor cells, enhances the infiltration of CD8^+^ and CD4^+^ T lymphocytes, and improves their antigen recognition of tumor cells—collectively strengthening the host immune system’s capacity to recognize and eliminate tumor cells (33–37). Additionally, radiotherapy stimulates tumor cells and stromal cells to secrete pro-inflammatory mediators and chemokines, which promote the immune infiltration of dendritic cells, macrophages, and T lymphocytes while increasing their cellular abundance, thereby effectively activating the tumor immune microenvironment (38–42). Radiotherapy can also indirectly upregulate the expression of immune checkpoints such as PD-L1 on tumor cell surfaces, in part through increased interferon-γ production (42, 43). Consequently, radiotherapy represents a potentially effective strategy for enhancing ICI efficacy.

Previous studies have shown that PD-L1 expression levels can significantly influence the efficacy of immunotherapy (44, 45). Subsequently, we analyzed the survival characteristics of patients with available PD-L1 expression data and explored the impact of PD-L1 expression on prognosis. After adjusting for intergroup differences by IPTW, we found that driver gene-negative NSCLC BM patients with PD-L1 expressing may achieve better PFS and IPFS benefits, which is consistent with previous studies. However, this association was not observed in OS, which may be attributed to the relatively small sample size due to missing PD-L1 expression data in some patients, as well as the heterogeneity of detection methods. This finding may also be attributed to the spatial and temporal heterogeneity of PD-L1 expression in tumors (46), which includes expression variability across different regions within the same tumor as well as expression differences between tumor lesions following recurrence, metastasis, or treatment (47, 48). Therefore, in clinical practice, attention should be directed toward the consistency of PD-L1 expression across various types of detection specimens in lung cancer, and analysis of PD-L1 expression patterns during tumor recurrence, metastasis, and post-treatment is essential. This approach will help enhance the accuracy and effectiveness of PD-L1 detection, which carries important clinical significance for guiding the application of immunotherapy in NSCLC (49).

In summary, this study demonstrates that brain radiotherapy combined with immunotherapy can provide clinical benefits for patients with driver gene-negative NSCLC BM. In particular, more significant clinical benefits from the combined therapy may be achieved in patients with fewer than two extracranial metastatic organs, a higher GPA score, and positive PD-L1 expression. However, this study also has several limitations. First, as an observational cohort study based on real-world data, despite application of IPTW to balance group differences, inherent selection bias and unmeasured confounding factors (e.g., socioeconomic status, physician preference) remain unavoidable. Moreover, in clinical practice, patients with better performance status and more favorable prognosis are more likely to receive ICI-containing therapy, which may bias our comparative outcomes. Second, due to undetermined PD-L1 expression levels in some patients, the sample size available for analysis was relatively small, which may have influenced the results. Third, Limited by its retrospective design and limited clinical data, this study only classified radiotherapy into SRS and WBRT, without stratifying the widely used WBRT plus local boost regimen. Future research should conduct more detailed subgroup analyses to investigate the efficacy of these specific radiotherapy modalities in patients with driver-negative NSCLC and BMs. Therefore, large-scale multicenter randomized controlled trials are warranted to further validate the conclusions of this study.

## 5 Conclusion

This study demonstrates that brain radiotherapy combined with immunotherapy and chemotherapy is effective and predictive in driver gene-negative NSCLC BM patients. In addition, SRS and PD-L1 expression may be associated with better prognosis in such patients. Consequently, the combination of radiotherapy and immunotherapy may serve as a promising novel clinical strategy for the management of driver gene-negative NSCLC BM.

## Data Availability

The original contributions presented in the study are included in the article/**Supplementary Material**. Further inquiries can be directed to the corresponding authors.
