# Supplementary material for: The effects of brain radiotherapy combined with immunotherapy and chemotherapy for driver gene-negative non-small-cell lung cancer with brain metastases
Source: Front Oncol. 2026 Jul 6;16:1763685. doi: 10.3389/fonc.2026.1763685 (PMC13381212; doi:10.3389/fonc.2026.1763685)
Supplement: Supplementary file 1 [file Supplementaryfile1.docx]

Supplementary File 1. Comparison of Baseline Characteristics: Systemic Therapy (Unadjusted IPTW)

| **Variable** | | **RT+CT^1^**  **(%)** | **RT+CT+ICI^1^**  **(%)** | **SMD^2^** |
| --- | --- | --- | --- | --- |
| Prop score | | - | - | 0.710 |
| Age≥65 | | 29.9 | 25.7 | 0.042 |
| Male | | 86.3 | 79.0 | 0.073 |
| Hypertension | | 20.5 | 26.7 | -0.062 |
| Diabetes | | 10.3 | 10.5 | -0.002 |
| Histopathology: LUSC | | 25.6 | 25.7 | -0.001 |
| No. of BMs ≤3 | | 59.8 | 57.1 | 0.027 |
| Clinical presentation: Symptomatic | | 46.2 | 58.1 | -0.119 |
| Temporal Heterogeneity: SBM | | 53.8 | 49.5 | 0.043 |
| SI | Heavy smoking | 45.3 | 54.3 | -0.090 |
|  | Light smoking | 10.3 | 5.7 | 0.045 |
|  | Moderate smoking | 2.6 | 3.8 | -0.012 |
|  | never smoke | 41.8 | 36.2 | 0.057 |
| ECOG | 0 | 2.6 | 3.8 | -0.012 |
|  | 1 | 88.9 | 87.6 | 0.013 |
|  | 2 | 8.5 | 8.6 | -0.000 |
| BMs lesion | All | 32.5 | 30.5 | 0.020 |
|  | Infratentorial BM | 17.1 | 18.1 | -0.010 |
|  | Supratentorial BM | 50.4 | 51.4 | -0.010 |
| T stage | 1 | 11.1 | 10.5 | 0.006 |
|  | 2 | 38.4 | 42.9 | -0.044 |
|  | 3 | 21.4 | 28.5 | -0.072 |
|  | 4 | 29.1 | 18.1 | 0.110 |
| N stage | 0 | 13.7 | 21.9 | -0.082 |
|  | 1 | 22.2 | 21.9 | 0.003 |
|  | 2 | 40.2 | 39.0 | 0.011 |
|  | 3 | 23.9 | 17.2 | 0.068 |
| No. of ECMs | ≥2 | 21.4 | 21.0 | 0.004 |
|  | 0 | 47.0 | 44.8 | 0.022 |
|  | 1 | 31.6 | 34.2 | -0.027 |
| ^1^Percentage of this variable's total count; ^2^Standardized Mean Difference (SMD).  Immune checkpoint inhibitors (ICI); chemotherapy (CT); brain radiotherapy (RT); Brain metastases (BMs); Smoking Index (SI); Eastern Cooperative Oncology Group (ECOG); Graded Prognostic Assessment (GPA); Lung adenocarcinoma (LUAD); Lung squamous cell carcinoma (LUSC); Extracranial metastasis (ECM); Programmed cell death 1 ligand 1(PD-L1); Tumor Proportion Score (TPS); Synchronous brain metastasis (SBM); Metachronous brain metastasis (MBM); whole-brain radiation therapy (WBRT); stereotactic radiosurgery (SRS). | | | | |
