# Supplementary figures and images for: The effects of brain radiotherapy combined with immunotherapy and chemotherapy for driver gene-negative non-small-cell lung cancer with brain metastases
Source: Front Oncol. 2026 Jul 6;16:1763685. doi: 10.3389/fonc.2026.1763685 (PMC13381212; doi:10.3389/fonc.2026.1763685)

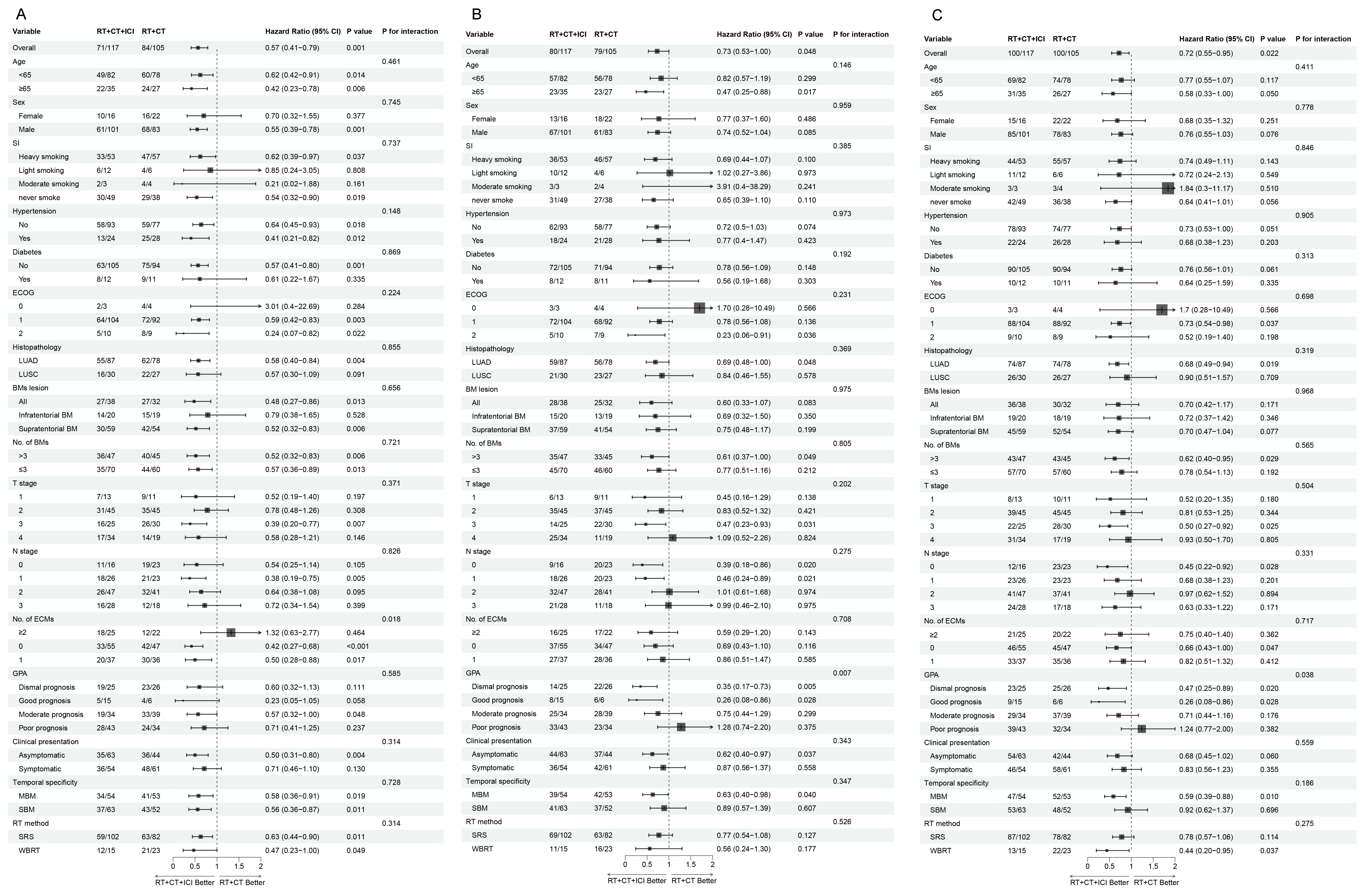

Supplement: Supplementary file 2 [file Supplementaryfile2.jpeg]
