# Supplementary material for: The effects of brain radiotherapy combined with immunotherapy and chemotherapy for driver gene-negative non-small-cell lung cancer with brain metastases
Source: Front Oncol. 2026 Jul 6;16:1763685. doi: 10.3389/fonc.2026.1763685 (PMC13381212; doi:10.3389/fonc.2026.1763685)
Supplement: Supplementary file 3 [file Supplementaryfile3.docx]

Supplementary File 3. Comparison of Clinical Characteristics: Radiotherapy Modalities (Unadjusted and Adjusted IPTW)

| **Variable** | | **Unadjusted IPTW** | | |  | **Adjusted IPTW** | | |
| --- | --- | --- | --- | --- | --- | --- | --- | --- |
|  |  | **SRS^1^**  **(%)** | **WBRT^1^**  **(%)** | **SMD^2^** |  | **SRS^1^**  **(%)** | **WBRT^1^**  **(%)** | **SMD^2^** |
| Prop score | | - | - | 0.976 |  | - | - | 0.124 |
| Age≥65 | | 27.7 | 28.9 | -0.012 |  | 27.6 | 25.5 | 0.021 |
| Male | | 82.6 | 84.2 | -0.016 |  | 83.1 | 80.9 | 0.022 |
| Hypertension | | 24.5 | 18.4 | 0.060 |  | 23.1 | 15.4 | 0.077 |
| Diabetes | | 11.4 | 5.3 | 0.061 |  | 10.4 | 7.5 | 0.029 |
| No. of BMs ≤3 | | 63.6 | 34.2 | 0.294 |  | 58.8 | 57.7 | 0.011 |
| Clinical presentation: Symptomatic | | 49.5 | 63.2 | -0.137 |  | 52.0 | 58.5 | -0.065 |
| Temporal Heterogeneity: SBM | | 51.6 | 52.6 | -0.010 |  | 52.1 | 55.4 | -0.033 |
| System therapy: RT+CT+ICI | | 55.4 | 39.5 | 0.160 |  | 52.9 | 55.9 | -0.029 |
| Histopathology: LUSC | | 26.1 | 23.7 | 0.024 |  | 26.1 | 23.2 | 0.029 |
| SI | Heavy smoking | 49.5 | 50.0 | -0.005 |  | 50.3 | 53.9 | -0.036 |
|  | Light smoking | 7.6 | 10.5 | -0.029 |  | 8.1 | 6.0 | 0.020 |
|  | Moderate smoking | 2.7 | 5.3 | -0.025 |  | 2.9 | 2.1 | 0.008 |
|  | never smoke | 40.2 | 34.2 | 0.060 |  | 38.7 | 38.0 | 0.008 |
| ECOG | 0 | 3.8 | 0.0 | 0.038 |  | 3.2 | 0.0 | 0.032 |
|  | 1 | 87.0 | 94.7 | -0.078 |  | 88.3 | 89.1 | -0.008 |
|  | 2 | 9.2 | 5.3 | 0.040 |  | 8.5 | 10.9 | -0.023 |
| BMs lesion | All | 28.3 | 47.4 | -0.191 |  | 31.5 | 32.9 | -0.014 |
|  | Infratentorial BM | 16.8 | 21.1 | -0.042 |  | 17.2 | 16.0 | 0.012 |
|  | Supratentorial BM | 54.9 | 31.5 | 0.233 |  | 51.3 | 51.1 | 0.001 |
| T stage | 1 | 9.2 | 18.4 | -0.092 |  | 10.5 | 16.2 | -0.057 |
|  | 2 | 39.7 | 44.7 | -0.051 |  | 40.7 | 42.0 | -0.014 |
|  | 3 | 24.5 | 26.3 | -0.019 |  | 24.9 | 27.1 | -0.021 |
|  | 4 | 26.6 | 10.6 | 0.161 |  | 23.9 | 14.7 | 0.092 |
| N stage | 0 | 15.8 | 26.3 | -0.106 |  | 17.3 | 22.2 | -0.048 |
|  | 1 | 21.7 | 23.7 | -0.019 |  | 22.6 | 22.7 | -0.000 |
|  | 2 | 40.2 | 36.8 | 0.034 |  | 39.4 | 36.8 | 0.025 |
|  | 3 | 22.3 | 13.2 | 0.091 |  | 20.7 | 18.3 | 0.023 |
| No. of ECMs | ≥2 | 21.7 | 18.5 | 0.033 |  | 21.0 | 18.5 | 0.025 |
|  | 0 | 46.2 | 44.7 | 0.015 |  | 46.1 | 43.2 | 0.029 |
|  | 1 | 32.1 | 36.8 | -0.048 |  | 32.9 | 38.3 | -0.053 |
| ^1^Percentage of this variable's total count; ^2^Standardized Mean Difference (SMD).  Immune checkpoint inhibitors (ICI); chemotherapy (CT); brain radiotherapy (RT); Brain metastases (BMs); Smoking Index (SI); Eastern Cooperative Oncology Group (ECOG); Graded Prognostic Assessment (GPA); Lung adenocarcinoma (LUAD); Lung squamous cell carcinoma (LUSC); Extracranial metastasis (ECM); Programmed cell death 1 ligand 1(PD-L1); Tumor Proportion Score (TPS); Synchronous brain metastasis (SBM); Metachronous brain metastasis (MBM); whole-brain radiation therapy (WBRT); stereotactic radiosurgery (SRS). | | | | | | | | |
