# Supplementary material for: The effects of brain radiotherapy combined with immunotherapy and chemotherapy for driver gene-negative non-small-cell lung cancer with brain metastases
Source: Front Oncol. 2026 Jul 6;16:1763685. doi: 10.3389/fonc.2026.1763685 (PMC13381212; doi:10.3389/fonc.2026.1763685)
Supplement: Supplementary file 4 [file Supplementaryfile4.docx]

Supplementary File 4. Comparison of Clinical Characteristics: PD-L1 expression (Unadjusted and Adjusted IPTW)

| **Variable** | | **Unadjusted IPTW** | | |  | **Adjusted IPTW** | | |
| --- | --- | --- | --- | --- | --- | --- | --- | --- |
|  |  | **PD-L1**  **<1%^1^**  **(%)** | **PD-L1**  **≥1%^1^**  **(%)** | **SMD^2^** |  | **PD-L1**  **<1%^1^**  **(%)** | **PD-L1**  **≥1%^1^**  **(%)** | **SMD^2^** |
| Prop score | | - | - | 1.189 |  | - | - | 0.043 |
| Age≥65 | | 31.3 | 34.0 | -0.026 |  | 32.3 | 29.3 | 0.030 |
| Male | | 86.6 | 83.0 | 0.035 |  | 82.7 | 83.7 | -0.010 |
| Hypertension | | 20.9 | 22.6 | -0.017 |  | 21.0 | 18.6 | 0.024 |
| Diabetes | | 10.4 | 13.2 | -0.028 |  | 12.2 | 9.6 | 0.026 |
| Histopathology: LUSC | | 20.9 | 24.5 | -0.036 |  | 18.7 | 20.2 | -0.015 |
| No. of BMs≤3 | | 56.7 | 67.9 | -0.112 |  | 59.7 | 56.2 | 0.034 |
| Clinical presentation: Symptomatic | | 43.3 | 50.9 | -0.077 |  | 46.3 | 38.7 | 0.076 |
| Temporal Heterogeneity: SBM | | 50.7 | 56.6 | -0.059 |  | 51.2 | 50.1 | 0.011 |
| System therapy: RT+CT+ICI | | 76.1 | 45.3 | 0.308 |  | 64.3 | 64.8 | -0.005 |
| Radiotherapy: WBRT | | 14.9 | 13.2 | 0.017 |  | 14.6 | 10.8 | 0.038 |
| SI | Heavy smoking | 52.2 | 50.9 | 0.013 |  | 53.5 | 56.7 | -0.033 |
|  | Light smoking | 7.5 | 11.4 | -0.039 |  | 8.4 | 9.4 | -0.009 |
|  | Moderate smoking | 6.0 | 1.9 | 0.041 |  | 4.2 | 3.2 | 0.010 |
|  | never smoke | 34.3 | 35.8 | -0.015 |  | 33.9 | 30.7 | 0.032 |
| ECOG | 0 | 3.0 | 0.0 | 0.030 |  | 1.7 | 0.0 | 0.017 |
|  | 1 | 86.6 | 94.3 | -0.078 |  | 90.6 | 94.7 | -0.040 |
|  | 2 | 10.4 | 5.7 | 0.048 |  | 7.7 | 5.3 | 0.023 |
| BMs lesion | All | 35.8 | 34.0 | 0.019 |  | 33.9 | 41.7 | -0.078 |
|  | Infratentorial BM | 20.9 | 13.2 | 0.077 |  | 16.7 | 13.8 | 0.029 |
|  | Supratentorial BM | 43.3 | 52.8 | -0.095 |  | 49.4 | 44.5 | 0.049 |
| T stage | 1 | 14.9 | 3.8 | 0.112 |  | 9.7 | 5.7 | 0.040 |
|  | 2 | 37.3 | 43.4 | -0.061 |  | 39.7 | 34.3 | 0.053 |
|  | 3 | 22.4 | 22.6 | -0.003 |  | 19.8 | 26.0 | -0.062 |
|  | 4 | 25.4 | 30.2 | -0.048 |  | 30.8 | 34.0 | -0.031 |
| N stage | 0 | 16.4 | 9.5 | 0.070 |  | 13.2 | 8.4 | 0.048 |
|  | 1 | 19.4 | 18.9 | 0.005 |  | 18.8 | 27.6 | -0.088 |
|  | 2 | 41.8 | 35.8 | 0.059 |  | 39.0 | 34.8 | 0.042 |
|  | 3 | 22.4 | 35.8 | -0.135 |  | 29.0 | 29.2 | -0.002 |
| No. of ECMs | ≥2 | 23.9 | 22.6 | 0.012 |  | 25.4 | 28.9 | -0.035 |
|  | 0 | 43.3 | 43.4 | -0.001 |  | 42.1 | 36.2 | 0.059 |
|  | 1 | 32.8 | 34.0 | -0.011 |  | 32.5 | 34.9 | -0.024 |
| ^1^Percentage of this variable's total count; ^2^Standardized Mean Difference (SMD).  Immune checkpoint inhibitors (ICI); chemotherapy (CT); brain radiotherapy (RT); Brain metastases (BMs); Smoking Index (SI); Eastern Cooperative Oncology Group (ECOG); Graded Prognostic Assessment (GPA); Lung adenocarcinoma (LUAD); Lung squamous cell carcinoma (LUSC); Extracranial metastasis (ECM); Programmed cell death 1 ligand 1(PD-L1); Tumor Proportion Score (TPS); Synchronous brain metastasis (SBM); Metachronous brain metastasis (MBM); whole-brain radiation therapy (WBRT); stereotactic radiosurgery (SRS). | | | | | | | | |
